# Retraction Note: The Arabidopsis NOT4A E3 ligase promotes PGR3 expression and regulates chloroplast translation

**DOI:** 10.1038/s41467-022-30354-z

**Published:** 2022-05-20

**Authors:** Mark Bailey, Aiste Ivanauskaite, Julia Grimmer, Oluwatunmise Akintewe, Adrienne C. Payne, Rory Osborne, Anne-Marie Labandera, Ross D. Etherington, Marjaana Rantala, Sacha Baginsky, Paula Mulo, Daniel J. Gibbs

**Affiliations:** 1 School of Biosciences, University of Birmingham, Edgbaston, B15 2TT UK; 2 Molecular Plant Biology, University of Turku, 20520 Turku, Finland; 3 Institute of Biochemistry and Biotechnology, Martin-Luther-University Halle-Wittenberg, Kurt-Mothes-Str. 3a, 06120 Halle, Saale Germany; 4 Present Address: Plant Sciences Department, Rothamsted Research, Harpenden, AL5 2JQ UK; 5 Present Address: Biochemistry of Plants, Faculty for Biology and Biotechnology, Ruhr-University Bochum, Universitätsstrasse 150, 44801 Bochum, Germany

**Keywords:** Ribosomal proteins, Ubiquitin ligases, Chloroplasts, Proteolysis in plants

Retraction to: *Nature Communications* 10.1038/s41467-020-20506-4, published online 11 Juanuary 2021.

In this Article, we reported that NOT4A ubiquitin-ligase was required for proper expression of PROTON GRADIENT REGULATION 3 (PGR3), chloroplast ribosome biogenesis, chloroplast protein translation and photosynthesis. However, it was since brought to our attention (by Hannes Ruwe and Christian Schmitz-Linneweber of Humboldt-Universität zu Berlin and Alice Barkan and Rosalind Williams-Carrier of the University of Oregon) that the Arabidopsis *not4a* T-DNA insertion line (*not4a*; GABI_134E03) used in the paper carries an additional mutation affecting the broader *PGR3* locus. We have independently confirmed the presence of a *PGR3* deletion in the genetic material used during the study. As a result, we can no longer unequivocally conclude that the reported *not4a* phenotypes can be attributed to loss of NOT4A rather than PGR3. For reasons that are currently unclear, the *PGR3* deletion was heterozygous in the *not4a* complementation lines (*N4A-G1* and *N4A-G3*) and as such, these lines do not serve as adequate controls. We consistently observe cosegregation of the *pgr3* deletion with the T-DNA insertion in *NOT4A*, despite being located on different chromosomes. While the basis for this apparent genetic linkage is currently unclear, this likely explains why the *pgr3* deletion was retained despite backcrossing the *not4a* insertion allele to the Col-0 wild type background as described in the Article. We thank Ruwe and colleagues for bringing the issues to our attention and we sincerely apologise for inadvertently misleading readers of the Article. All authors agree with retraction of the Article.

